# Pre-operative magnetic resonance imaging can predict prostate cancer with risk for positive surgical margins

**DOI:** 10.1007/s00261-022-03543-z

**Published:** 2022-05-16

**Authors:** M. Quentin, L. Schimmöller, T. Ullrich, B. Valentin, D. Demetrescu, R. Al-Monajjed, D. Mally, I. Esposito, P. Albers, G. Antoch, C. Arsov

**Affiliations:** 1 Medical Faculty, Department of Diagnostic and Interventional Radiology, University Dusseldorf, Moorenstr. 5, 40225 Dusseldorf, Germany; 2 Medical Faculty, Department of Urology, University Dusseldorf, 40225 Dusseldorf, Germany; 3 Medical Faculty, Institute of Pathology, University Dusseldorf, 40225 Dusseldorf, Germany

**Keywords:** Prostate cancer, Prostate MRI, Staging, Prostatectomy, Positive surgical margins, Predictive biomarker

## Abstract

**Purpose:**

Analysis of patients with pre-operative 3 T multiparametric prostate MRI (mpMRI) to determine reliable MRI-based risk predictors of patients at risk for positive surgical margins (PSM) in robotic assisted radical prostatectomy (RPE).

**Methods:**

Consecutive patients with 3 T mpMRI and subsequent RPE from 01/2015 to 12/2018 were retrospectively included. Patients were compared regarding clinical and MRI related parameters such as length of capsular tumor contact (LCC) and distance to the membranous urethra (UD).

**Results:**

Forty-nine of 179 patients (27%) had PSM in 70 different localizations, with the majority located at the capsule (57%, 40/70), mostly apical and/or posterior. The second most often PSM occurred at the apical urethra (22%, 15/70). PCA was visible on mpMRI at the localization of PSM in 93% at the capsule and in 80% at the urethra. PSA, PI-RADS classification, extraprostatic extension (EPE), and seminal vesicles infiltration (SVI) on MRI were significantly higher / more frequent in patients with PSM. LCC (AUC 0.710), EPE (AUC 0.693), and UD (1-AUC 0.673) predicted PSM (overall). An UD of ≤ 3.5 mm showed the highest accuracy of 95% (J = 0.946) for PSM at the urethra and a LCC of ≥ 22.5 mm with 77% (*J* = 0.378) for PSM at the capsule.

**Conclusion:**

PSM occurred mostly in the apex and/or posteriorly at the capsule or at the apical urethra. LCC was the best MRI predictor for PSM at the capsule and UD for tumors with PSM at the apical urethra. Using these MRI parameters readers might pre-operatively determine PCA localizations at risk for PSM.

**Supplementary Information:**

The online version contains supplementary material available at 10.1007/s00261-022-03543-z.

## Introduction

Multiparametric prostate MRI (mpMRI) plays an increasingly significant role in prostate cancer (PCA) diagnostics. Various studies showed that mpMRI is highly sensitive in visualization of PCA suspect lesions and targeted MR-guided biopsy better predict the correct Gleason score on radical prostatectomy (RPE) [1, 2]. Apart from the detection, mpMRI comprises various staging information and is currently seen as the best available imaging tool for assessing extraprostatic extension (EPE) and the T-stage [3–5]. Urologic assessment to predict nonorgan-confined PCA was limited to nomograms based on biopsy results [6]. Meta-analysis showed a moderate sensitivity of 61% of MRI to detect EPE (overall stage T3 disease) with high specificity of 88%, but at 3 Tesla (3 T) MRI also sensitivities above 70% are reported [3, 4]. Over the last year’s positron emission tomography (PET) with a prostate specific membrane antigen (PSMA) tracer got higher priority focusing primarily biochemical PCA recurrence and/or M and N staging in high-risk PCA [7–9]. Additional improvement might be achieved by PSMA-PET/MRI and/or artificial intelligence, respectively radiomics [10]. The European Association of Urology (EAU) Guidelines recommend to use pre-biopsy mpMRI for local staging [11].

MRI directed intraoperative frozen-section analysis during nerve-sparing RPE have been shown to reduce the rate of positive surgical margins (PSM) by repeat excision of the tumor at the potential sites of EPE [12]. Nevertheless, PSM also occur in patients with organ-confined PCA. These patients have a higher recurrence rate compared to patients with organ-confined PCA or focal (microscopic) EPE with negative margins [13]. The likelihood of PSM is strongly related with the surgeon’s experience independent of the surgical approach [14]. However, while avoiding PSM is the primary goal of RPE, sparing the neurovascular bundles and the membranous urethra is important for continence and potency. Beyond tumor detection and staging, mpMRI provides additional information on anatomy, prostate volume, and tumor localization which could help to identify areas at risk for PSM [15].

Aim of this study was to analyze 3 T mpMRI examinations of patients with subsequent RPE to determine reliable MRI-based risk predictors to identify patients at risk for PSM. Therefore, PCA visibility in PSM localization, length of capsular contact (LCC), EPE, distance to the membranous urethra (UD), and tumor infiltration in other structures was analyzed. The results are supposed to help radiologists to focus on relevant risk predictors and to help urologists with pre-operative planning to avoid PSM.

## Materials and methods

### Study design

We retrospectively analyzed all consecutive patients from January 2015 to December 2018 with in-house robotic assisted RPE and prior mpMRI for PCA detection (*n* = 179) in this single-center cohort study. We did not include patients without or incomplete mpMRI (Supp. Fig. S1). None of the patients had received prior treatment for PCA. The study was approved by the local Independent Ethics Committee (IEC) (Study number: 2018-227-RetroDEuA; Medical Faculty, University Dusseldorf).

### Imaging

The mpMRI of the prostate was performed on a 3 T MRI scanner (Magnetom TIM Trio™, Prisma™ or Skyra™; Siemens Healthineers) with 18 or 60 channel phased-array surface-coils plus/minus 32 channel spine coil according to the European Society of Urogenital Radiology (ESUR) guidelines and the national recommendations [16, 17]. The MR protocol contained T1-weighted images (from the whole pelvis), T2-weighted images (in 3 planes, 3 mm slice thickness), diffusion-weighted images (3 mm slice thickness, b-values: 0, 500, 1000 s/mm^2^ for ADC calculation, an additional high b-value ≥ 1400 s/mm^2^), and dynamic contrast-enhanced images (DCE; 3 mm slice thickness, scan time 3 min, temporal resolution < 9 s). The detailed MR-protocol has been published previously [18]. All patients received butylscopolamine (20 mg Buscopan®, Boehringer Ingelheim Pharma) to suppress bowel peristalsis.

### Image analysis

The mpMRI was retrospectively analyzed by two experienced, board-certified radiologists in consensus subspecialized in prostate MRI (M.Q. and LS, both with more than 10 years’ experience in reading prostate MRI) regarding the following aspects in relation to the histopathologic examination of the prostatectomy specimens (localization of positive surgical margins): visible PCA, extraprostatic extension (EPE), length of capsular tumor contact (LCC), PCA distance to the membranous urethra (UD), and seminal vesicle infiltration (SVI). All measurements were performed on high resolution coronary, sagittal, and/or axial T2-weighted images using the plane that showed the lesion best with reference to DWI (ADC map and high b-value images) and DCE. EPE was defined as extension of the tumor beyond the gland boundary (in mm). LCC was determined by the greatest capsular tumor contact in the different planes using curvilinear measurements along the capsule (in mm). UD was defined as shortest distance of the tumor to the proximal membranous urethra, located between the apex of the prostate and the bulb of the corpus spongiosum, extending through the urogenital diaphragm (in mm). SVI was evaluated if the tumor showed a measurable infiltration of the SV (in mm). PI-RADS classification was performed retrospectively in consensus using PI-RADS version 2.1. Radiologists were blinded to clinical parameters especially PCA aggressiveness, tumor stage, and PSA values.

### Prostatectomy

Robotic assisted radical prostatectomy (RPE) was performed with a 3-arm Da Vinci Surgical Si System (Intuitive Surgical) by three different board-certified surgeons (with each more than 10 years’ experience) and a transperitoneal access. The decision for curative surgery, as well as decisions for lymph node dissection, was made according to the guidelines of the European Association of Urology [11]. Histopathology was performed according to the recommendations of the International Society of Urological Pathology (ISUP).

### Statistical analysis

Descriptive statistics were used to present patient characteristics. Statistical analyses were performed using IBM SPSS® Statistics (Version 21, IBM Deutschland GmbH). Data are expressed as mean ± SD and median + IQR. Nonparametric Mann–Whitney-U test was used to compare the group of patients with and without PSM. Statistical significance was defined as a *p*-value < 0.05. Receiver operating characteristic (ROC) analyses were used for quantifying the impact of clinical and MRI related predictors. Youden index (J = sensitivity + specificity − 1) was used to measure the clinical diagnostic ability of LCC and UD.

## Results

### Study population

From 179 included patients, 49 patients had PSM (R1) in at least one localization. The time between MRI and RPE was in mean 12 ± 7.3 weeks. PSA was significant higher in patients with PSM compared to patients without PSM (Table 1). Clinical and postoperative parameters showed limited ability to assess the risk of PSM: age (AUC 0.507; *p* = 0.879), prostate volume (AUC 0.549; *p* = 0.315), PSAD (AUC 0.597; *p* = 0.05), PSA (AUC 0.633; *p* = 0.006), ISUP grade in biopsy (AUC 0.554; *p* = 264), ISUP grade after prostatectomy (AUC 0.597; *p* = 0.05), with postoperative T-stage being the best parameter (AUC 0.681; *p* < 0.001).

**Table 1:** Patient baseline characteristics

		NSM	PSM	*P*-value*
Clinical data	**Patients**; number	130	49	
	**Age** in y; mean ± SD	66 ± 8.08	66 ± 7.95	0.879
	**PSA value** in ng/ml; median (IQR)	8.84 (6.29 – 12.79)	11 (7.60 – 15.17)	**0.006**
	**PSAD** in ng/ml/cm^3^; median (IQR)	0.23 (0.15 – 0.37)	0.27 (0.22 – 0.40)	0.046
	**Prostate volume** in ml; median (IQR)	36 (29 – 51)	38 (32 – 50)	0.315
	**ISUP; post-biopsy** median (IQR)	2 (2 – 3.75)	2 (2 – 4)	0.239
RPE	**ISUP; post-surgery** median (IQR)	2 (2 – 3)	3 (2—4)	**0.031**
	**T2a-c**, % (n)	65% (85)	35% (17)	** < 0.001**
	**T3a**, % (n)	22% (28)	24% (12)	
	**T3b**, % (n)	13% (17)	41% (20)	

*Mann–Whitney-U test

*NSM* negative surgical margins, *PSM* positive surgical margins*, PSA* prostate specific antigen, *PSAD* prostate specific antigen density, *ISUP* International Society of Urological Pathology Grade Group

Bold values indicate *P*-value < 0.05

### Characterization of PSM

PSM were documented in 70 different localizations. The majority of PSM (57%) were located at the capsule, and therein mostly apical and posteriorly (Table 2). The apical urethra was the most infiltrated structure (22%). MpMRI showed a high ability to visualize PCA at the localization of PSM, ranging from 80% at the urethra to 100% at the bladder neck. In 6 patients with PSM the tumor was invisible on mpMRI in the following localizations: 3 at the capsule, one at the seminal vesicles, and in 2 patients at the urethra.

**Table 2:** Characterization of lesions with positive surgical margins (PSM)

in % (n)				PSM
RPE	PSM localization	Side	Left	39% (27/70)
			Right	24% (17/70)
			Both	37% (26/70)
		Capsule	All	57% (40/70)
			Basal	18% (7/40)
			Midgland	27% (11/40)
			Apical	55% (22/40)
			Anterior	35% (14/40)
			Posterior	43% (17/40)
			Lateral	22% (9/40)
		Urethra, apical		22% (15/70)
		SV		14% (10/70)
		Bladder neck		7% (5/70)
MRI	visible PCA in PSM localization	Capsule	all	93% (37/40)
			LCC 23 ± 12.5 mm (mean ± SD)	
			EPE	68% (25/37)
			EPE 4.4 ± 2.1 mm (mean ± SD)	
		Urethra, apical		80% (12/15)
		SV		90% (9/10)
		Bladder neck		100% (5/5)

*PSM* positive surgical margins, *RPE* radical prostatectomy, *SV* seminal vesicles, *EPE* extraprostatic extension, *LCC* length of capsular contact of tumor

### Prediction of PSM

**Fig. 1:**
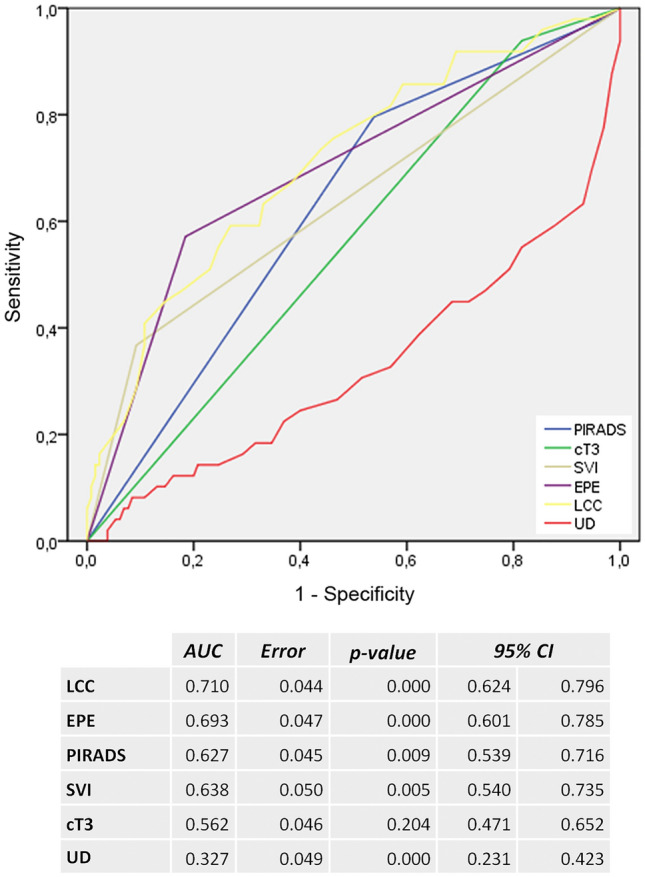
ROC analysis of MRI related predictors for positive surgical margins (PSM). *cT3* clinical T3 stage, *LCC* length of capsular contact of tumor, *EPE* extraprostatic extension, *PI-RADS* Prostate Imaging Reporting and Data System v2.1, *SVI* seminal vesicles infiltration, *UD* PCA distance to the membranous urethra

**Fig. 2:**
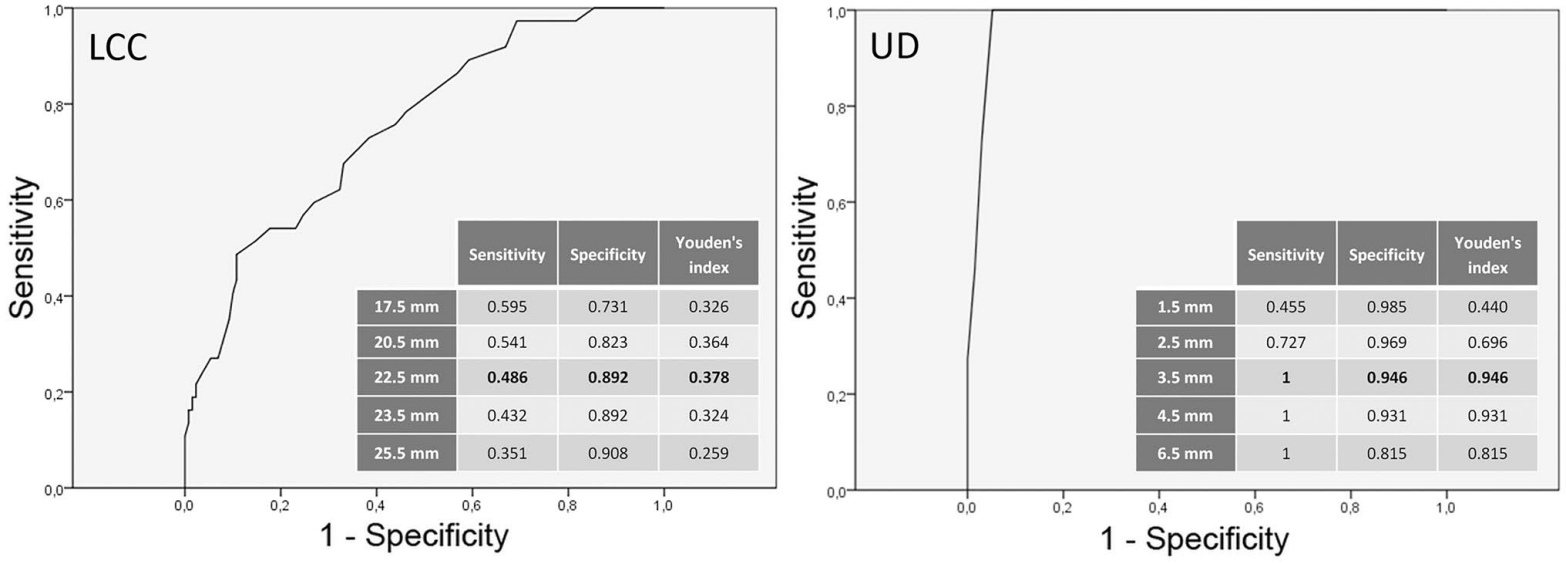
ROC analysis of LCC as predictor for PSM at the capsule and UD as predictor for positive surgical margin (PSM) at the apical urethra. *LCC* length of capsular contact of tumor, *UD* PCA distance to the membranous urethra

**Fig. 3:**
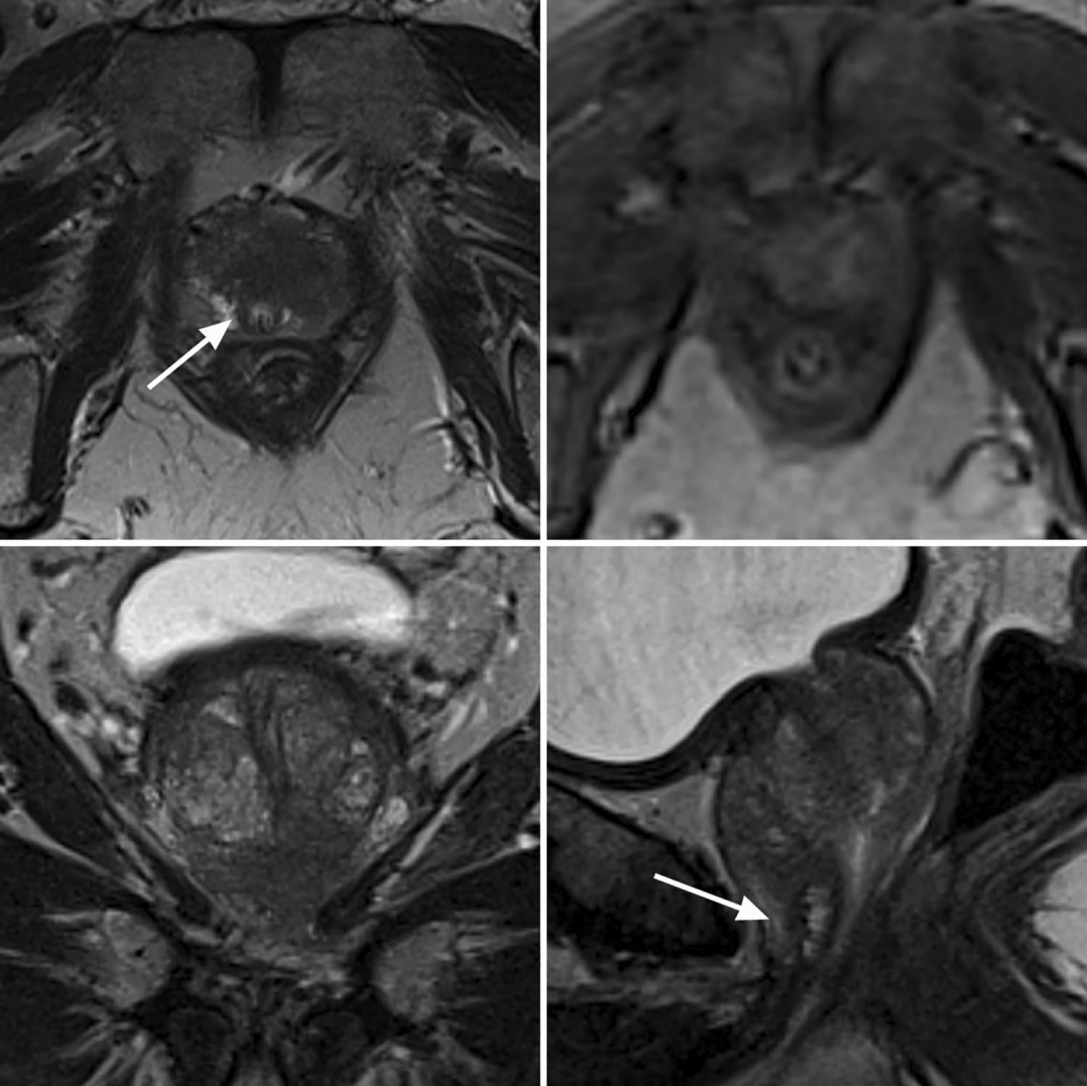
Patient with organ-confined prostate cancer (T2c) and positive surgical margins at the apical urethra with broad tumor contact to the anterior circumference of the apical urethra on mpMRI. PCA distance to the membranous urethra (UD) was 2 mm (arrows). Upper left: T2 axial, upper right: *DCE* lower left, T2 coronal, lower right: T2 sagittal

**Fig. 4:**
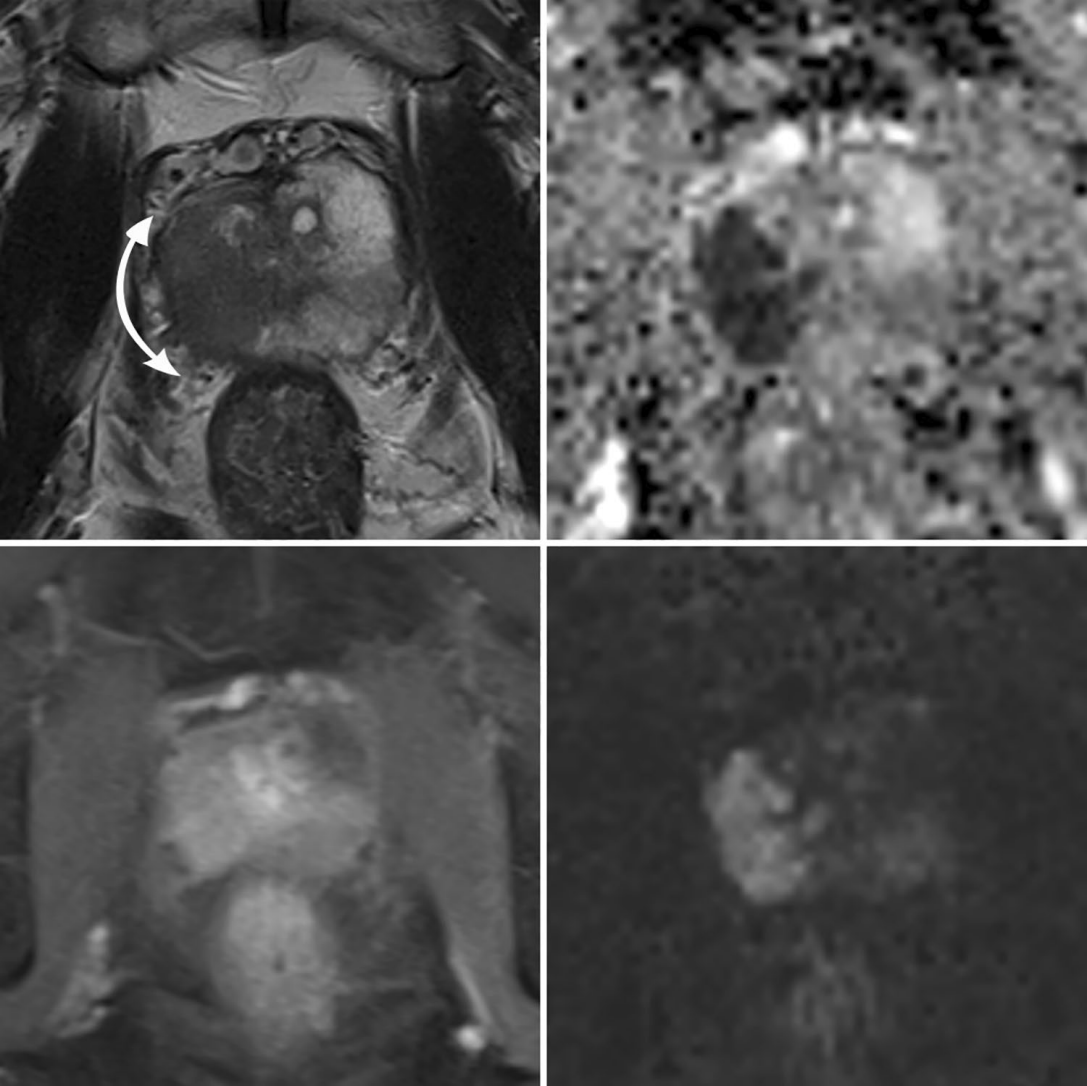
Patient with extraprostatic extension posterolateral left on mpMRI (T3a) and positive surgical margins in this localization. The length of capsular contact (LCC) of the PCA was 38 mm (double-headed arrow) and extraprostatic extension (EPE) 4 mm. Upper left: T2 axial, upper right: ADC map, lower left: DCE, lower right: high b-value image (b2000)

PI-RADS scores were significantly higher in patients with PSM. Also, extraprostatic extension and seminal vesicles infiltration on MRI was significantly more frequent in patients with PSM (Table 3). Index lesions of these patients had a significant higher LCC and a significant lower UD. LCC was the best MRI parameter to predict PSM (AUC 0.710). AUC values for the other MRI parameters were as follows: EPE 0.693, UD 0.327, SVI 0.638, PI-RADS 0.627, and cT3 stage 0.562 (Fig. 1). In the cases with PSM at the apical urethra 1-AUC for UD was 0.981 (*p* < 0.001). A UD ≤ 3.5 mm showed highest accuracy with a sensitivity of 100% and specificity of 95% (*J* = 0.946) for PSM; a LCC of ≥ 22.5 mm with a sensitivity of 49% and specificity of 89% (*J* = 0.378) for PSM at the capsule (Figs. 2, 3 and 4).

**Table 3:** MRI prediction parameters for positive surgical margins (PSM)

			NSM	PSM	*P*-value*
PI-RADS v2.1% (n)		3	3% (4/130)	2% (1/49)	**0.002**
		4	43% (56/130)	18% (9/49)	
		5	54% (70/130)	80% (39/49)	
cT3% (n)			72% (107/130)	94% (46/49)	**0.040**
EPE % (n)			18% (24/130)	57% (28/49)	** < 0.001**
EPE in mm; median (IQR)			3 mm (2 – 5)	4 mm (3 – 6)	
SVI % (n)			9% (12/130)	37% (18/49)	** < 0.001**
SVI in mm; median (IQR)			7 mm (5.25 – 11)	8 mm (6 – 11)	
Index lesion	Localization	basal	27% (35/130)	29% (14/49)	0.235
		midgland	33% (43/130)	45% (22/49)	
		apical	40% (52/130)	26% (13/49)	
		anterior	30% (39/130)	37% (18/49)	0.143
		posterior	34% (44/130)	41% (20/49)	
		lateral	36% (47/130)	22% (11/49)	
	LCC in mm; median (IQR)		12 (8 – 18)	20 (13 – 26) Cases with PSM at the capsule: 22 (14–28)	** < 0.001**
	UD in mm; median (IQR)		15 (12 – 27)	8 (3 – 15) Cases with PSM at the urethra: 2 (1–3)	** < 0.001**

*Mann–Whitney-U test

*NSM* negative surgical margins, *PSM* positive surgical margins, *EPE* extraprostatic extension, *SVI* seminal vesicle infiltration, *LCC* length of capsular contact of tumor, *UD* distance to the membranous urethra, *cT3* clinical T3 stage

Bold values indicate *P*-value < 0.05

## Discussion

MpMRI (3 T) showed a high ability to visualize PCA at the localization of PSM, which occurred mostly apical and/or posteriorly at the capsule or at the apical urethra. In 6 patients with PSM the tumor was invisible on mpMRI. 35% of patients with PSM had organ-confined PCA. Best clinical parameter to predict PSM was the postoperative T-stage. LCC was the best MRI predictor for PSM at the capsule, which performed better than the clinical parameters. Nevertheless, in tumors with PSM at the apical urethra, UD was the best MRI parameter. Highest accuracy was documented for UD ≤ 3.5 mm, indicating a high risk for PSM at the urethra and for LCC ≥ 22.5 mm, indicating a high risk for PSM at the capsule. Using these MRI parameters PCA localizations at risk for PSM might be pre-operatively determined.

Park et al. developed and validated a scoring system for MRI to predict PSM inducing the PI-RADS score, tumor location on posterolateral side or at the apex, and length of capsular contact, archiving an AUC value of 0.80 [19]. Compared to our results the score was slightly better than LCC alone, but inferior to UD for apical tumors. There are some aspects of this score that needs to be discussed. First, PI-RADS score was weighted more heavily compared to tumor localization. Second, PI-RADS category 3 (clinically significant cancer is equivocal) and PI-RADS category 4 (clinically significant is likely to be present) have the same impact on the score, although in clinically consequence is different. Third, capsular contact ≤ 14 mm was rated with 0. Other studies showed that the risk for EPE was already increased at LCC ≥ 11 mm [4]. Main limitation is that risk factors for PSM depend on the localization of PSM and therefore should be assessed separately for either risk of PSM at the apical urethra or at the capsule.

The diagnostic performance of mpMRI is influenced by the different prevalence of EPE in different risk stratified cohorts [20]. High negative predictive values (88%) are only reached in low-risk cohorts, where patients could benefit if they were selected for nerve sparing surgery by the prior mpMRI. Positive predictive value was highest (89%) in high-risk cohort, which could help to reduce the risk of PSM. In a prospective randomized single-center trial preoperative MRI could only reduce PSM in low-risk PCA [21]. According to the authors a main limitation is lacking communication between radiologists and urologists, which is crucial to adopt surgical approaches.

A recent meta-analysis showed that mpMRI had a considerable impact on the extent of resection during RPE, but modifications of neuro-vascular-bundle preservation did not influence PSM rates [22]. However, apart from relatively small number of studies, mostly retrospective design, different MRI protocols, scanner, and field strength, a further reason for these results might be the lack of standardized MRI reading.

This study is limited by the retrospective design and the single-center evaluation. Although PCA is usually a slow growing tumor, the time interval between MRI and operation might have influenced the results. MRI was acquired at 3 T scanners and reading was performed by subspecialized experts in consensus, but we did not access interreader variability, so less experienced readers may perform differently. Furthermore, PSM at the capsule and at the urethra may have different clinical impact, therapeutic consequence, and risk for biochemical recurrence (BCR). However, this study focuses on the MRI visibility and prediction of PSM.

In conclusion, mpMRI (3 T) was an excellent tool to visualize PCA and could help to identify patients at risk for PSM, which occurs mostly apical and/or posteriorly at the capsule or at the apical urethra. Best predictive parameters were LCC at the capsule and UD for apical located tumors. Since communication with the surgeon is crucial to adopt the surgical strategy, EPE, LCC and UD should be highlighted in the structured report.

## Supplementary Information

Below is the link to the electronic supplementary material.

Supplementary file1 (TIF 5882 kb)

## Abbreviations

LCC: Length of capsular tumor contact
EPE: Extraprostatic extension
mpMRI: Multiparametric prostate MRI
PCA: Prostate cancer
PI-RADS: Prostate Imaging Reporting and Data System
PSM: Positive surgical margins
SVI: Seminal vesicle infiltration
UD: PCA distance to the membranous urethra
