# Supplementary figures and images for: Pre-operative magnetic resonance imaging can predict prostate cancer with risk for positive surgical margins
Source: Abdom Radiol (NY). 2022 May 16;47(7):2486–93. doi: 10.1007/s00261-022-03543-z (PMC9226088; doi:10.1007/s00261-022-03543-z)

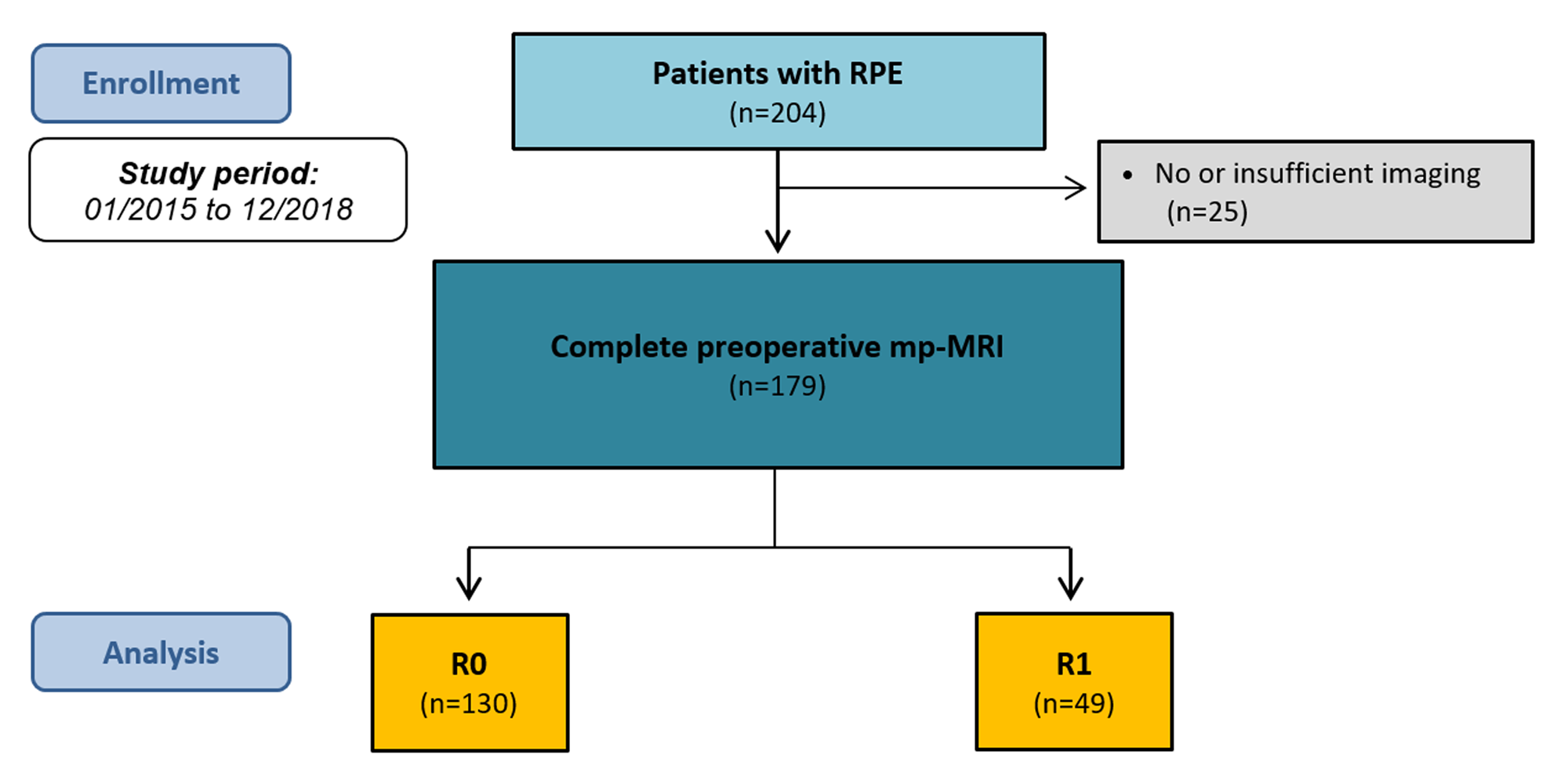

Supplement: Supplementary file 1 — Supplementary file1 (TIF 5882 kb) [file 261_2022_3543_MOESM1_ESM.tif]
